# Supplementary material for: Deep-Learning-Based Prediction of t(11;14) in Multiple Myeloma H&E-Stained Samples
Source: Cancers (Basel). 2025 May 22;17(11):1733. doi: 10.3390/cancers17111733 (PMC12153534; doi:10.3390/cancers17111733)
Supplement: Supplementary file 1 [file cancers-17-01733-s001.zip › cancers-3622474-supplementary.pdf]

Supplementary file

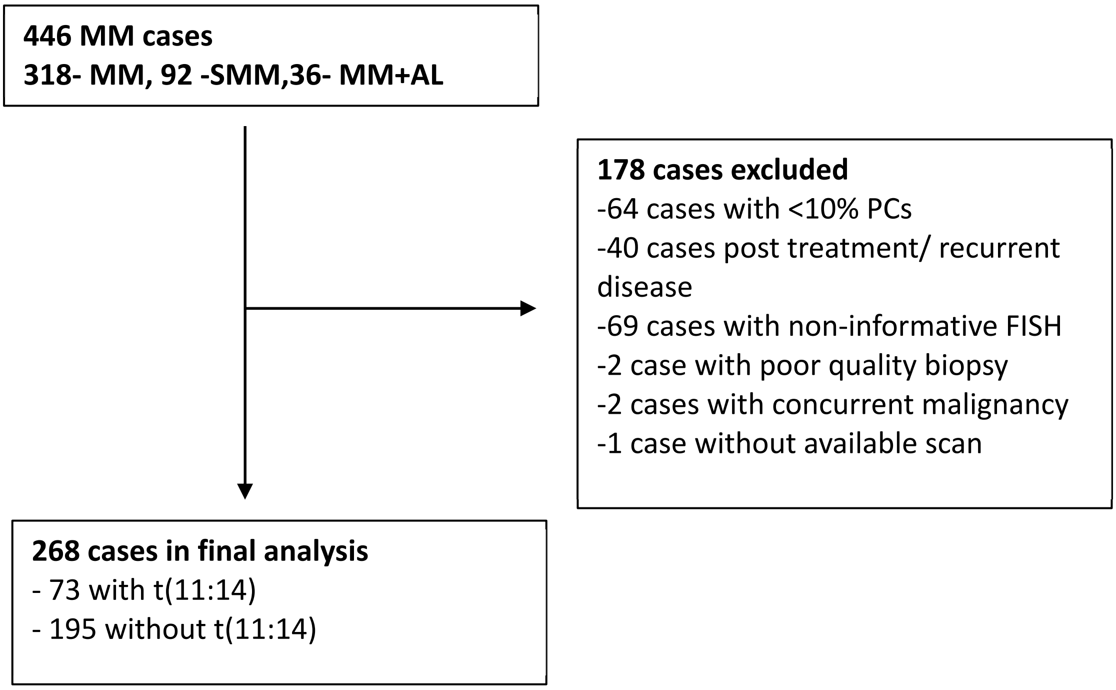

AL- amyloidosis; MM- multiple myeloma; SMM – smoldering multiple myeloma

Figure S1. Patient Disposition.

Table S1. Univariate analysis of factors associated with conclusive AI results.

	**Entire cohort (n=268)**	**Conclusive results for t(11;14)(n=102, 38.1%)**	**Inconclusive results for t(11;14) (n=166, 61.9%)**	**p Value**
t(11;14) positive, n (%)	73 (27.2)	25 (24.5)	48 (28.9)	0.431
Age years, median (range)	69.9 (41.8-92.1)	70 (44.3-92.1)	69.1 (41.8-89.7)	0.241
Sex, Males, n (%)	147 (54.9)	55 (53.9)	92 (55.4)	0.811
PC Dyscrasia Type, n (%)	-			
Active MM	196 (73.1)	85 (83.3)	111 (66.9)	
Smoldering MM	47 (17.5)	11 (10.8)	36 (21.7)	0.006
Active MM with AL	22 (8.2)	4 (3.9)	18 (10.8)	
Other*	3 (1.1)	2 (2)	1 (0.6)	
% of PCs in BMB, median (IQR)	30 (20-60)	50 (20-70)	25 (20-50)	0.0002
Calcium mg/dL, median (IQR)	9.4 (9-9.9)	9.4 (9-9.8)	9.4 (9-9.9)	0.797
Hypercalcemia[‡], n (%)	17 (7)	5 (5.3)	12 (8.1)	0.396

Creatinine mg/dL, median (IQR)	0.99 (0.79-1.38)	1.04 (0.8-1.48)	0.97 (0.77-1.3)	0.357
Renal insufficiency$^{\alpha}$, n (%)	29 (11.4)	13 (13)	16 (10.4)	0.523
Hemoglobin mg/dL, mean (SD)	11.4 (2)	11 (2.12)	11.7 (1.88)	0.006
Anemia$^{\beta}$, n (%)	61 (23.7)	30 (30)	31 (19.7	0.06
Lytic bone lesions, n (%)	128 (50.6)	61 (62.2)	67 (43.2)	0.003
Heavy Chain Subtype, n (%)	-			0.309
IgG	126 (49)	54 (52.9)	72 (46.5)	
Non IgG	131 (51)	48 (47.1)	83 (53.5)	
Light Chain Subtype, n (%)	-			0.304
Kappa	173 (65)	70 (70)	103 (62)	
Lambda	92 (34.6)	30 (30)	62 (37.3)	
Nonsecretory	1 (0.4)	0 (0)	1 (0.6)	
FLCr, median (IQR)	55 (8.4-214)	84 (9.4-280)	46 (7.9-172.5)	0.039
Number of CA, median (IQR)	1 (0-2)	1 (0-2)	1 (0-2)	0.94
Any Other CA, n (%)	25 (9.3)	14 (13.7)	11 (6.6)	0.052
High Cytogenetic Risk$^{\gamma}$, n (%)	109 (40.7)	41 (40.2)	68 (41)	0.901
Disease stage- ISS, n (%)	-			0.489
I	77 (42.3)	28 (38.4)	49 (45)	
II	45 (24.7)	20 (27.4)	25 (22.9)	
III	60 (33)	25 (34.2)	35 (32.1)	
Missing, n	86	29	57	
Slide Age, years, median (IQR)	3.1 (1.9-4.3)	3.1 (2.2-4.7)	3 (1.8-4.1)	0.104

** Includes plasma cell leukemia and solitary plasmacytoma*

‡ *Serum calcium >11 mg/dL*

$^{\alpha}$ *Serum creatinine >2mg/dL*

$^{\beta}$ *Hemoglobin of <10 g/dL*

$^{\gamma}$ *High risk cytogenetics was determined as positive for t(4;14), t(14;16), t(14;20), del(17p) and gain 1q.*

AL- Light Chain Amyloidosis; BM- Bone marrow; BMB- Bone marrow biopsy; CA- Cytogenetic Abnormalities; FLCr- Free Light Chain Ratio; ISS- International Staging System; IQR- Interquartile Range; MM- Multiple Myeloma; PC- Plasma cell; R-ISS- Revised International Staging System; SD- Standard Deviation
